# A Retrospective Study of the Diagnostic Accuracy of In Vivo Reflectance Confocal Microscopy for Basal Cell Carcinoma Diagnosis and Subtyping

**DOI:** 10.3390/jcm8040449

**Published:** 2019-04-03

**Authors:** Mihai Lupu, Iris Maria Popa, Vlad Mihai Voiculescu, Daniel Boda, Constantin Caruntu, Sabina Zurac, Calin Giurcaneanu

**Affiliations:** 1 Department of Dermatology, “Carol Davila” University of Medicine and Pharmacy, 050474 Bucharest, Romania; lupu.g.mihai@gmail.com (M.L.); voiculescuvlad@yahoo.com (V.M.V.); calin.giurcaneanu@gmail.com (C.G.); 2 Department of Plastic and Reconstructive Surgery, “Bagdasar-Arseni” Clinical Emergency Hospital, 041915 Bucharest, Romania; irismpopa@gmail.com; 3 Department of Dermatology, “Elias” University Emergency Hospital, 011461 Bucharest, Romania; 4 Department of Dermatology, “Prof. N. Paulescu” National Institute of Diabetes, Nutrition and Metabolic Diseases, 011233 Bucharest, Romania; daniel.boda@yahoo.com; 5 Department of Physiology, “Carol Davila” University of Medicine and Pharmacy, 050474 Bucharest, Romania

**Keywords:** Carcinoma, basal cell, dermoscopy, microscopy, confocal, retrospective studies, skin neoplasms

## Abstract

Current national and European guidelines recommend distinct management approaches for basal cell carcinoma (BCC) based on tumor location, size, and histopathological subtype. In vivo reflectance confocal microscopy (RCM) is a non-invasive skin imaging technique which may change the diagnostic pathway for BCC patients. This study aimed to determine the sensitivity and specificity of RCM for BCC diagnosis, assess the predictive values of several confocal criteria in correctly classifying BCC subtypes, and evaluate the intraobserver reliability of RCM diagnosis for BCC. We conducted a retrospective study in two tertiary care centers in Bucharest, Romania. We included adults with clinically and dermoscopic suspect BCCs who underwent RCM and histopathological examination of excision specimens. For RCM examinations, we used the VivaScope 1500 and histopathology of the surgical excision specimen was the reference standard. Of the 123 cases included in the analysis, BCC was confirmed in 104 and excluded in 19 cases. RCM showed both high sensitivity (97.1%, 95% CI (91.80, 99.40)) and specificity (78.95%, 95% CI (54.43, 93.95)) for detecting BCC. Several RCM criteria were highly predictive for BCC subtypes: cords connected to the epidermis for superficial BCC, big tumor islands, peritumoral collagen bundles and increased vascularization for nodular BCC, and hyporefractile silhouettes for aggressive BCC. Excellent intraobserver agreement (κ = 0.909, *p* < 0.001) was observed. This data suggests that RCM could be used for preoperative diagnosis and BCC subtype classification in patients with suspected BCCs seen in tertiary care centers.

## 1. Introduction

Basal cell carcinoma (BCC) is the most prevalent skin cancer worldwide. In Europe, BCC incidence has been constantly rising by approximately 5% annually over recent decades [1], causing a major burden on healthcare systems [2,3]. Adding to this, an abrupt increase in BCC incidence in the young population is reported [4,5,6]. Although BCC mortality is low (0.0028% to 0.55%) [7], these tumors are locally invasive and can induce significant morbidity owing to their frequent development on the head and neck.

Current national and European guidelines [8,9] advise distinct therapeutic approaches of BCC based on tumor location, size, and histopathological subtype. With the increasing number of efficient non-surgical treatment options for superficial BCC (sBCC) [10], the histopathological subtype becomes of special interest in choosing the most appropriate management course [11,12,13,14]. Guidelines also recommend BCC diagnosis confirmation and histological subtyping through a punch biopsy [15,16]. However, a punch biopsy fails to diagnose an aggressive BCC subtype in one out of six tumors [17]. Therefore, histopathological examination of the entire tumor specimen remains the most accurate approach of establishing BCC histopathological subtype [18,19,20].

The preoperative assessment of BCC histological subtypes through non-invasive techniques may reduce the number of painful invasive diagnostic procedures, lower the delay between diagnosis and treatment, and lower the burden on healthcare systems through reducing administrative workloads and financial costs [21,22]. Several recent studies have tried to correlate dermoscopic criteria to certain BCC subtypes [23,24], without consistent results.

High resolution non-invasive skin imaging may change the diagnostic pathway in the case of BCC patients [25,26]. In vivo reflectance confocal microscopy (RCM) is a novel, non-invasive imaging technique capable of producing horizontal optical sections of the skin [27]. RCM enables examination of the entire lesion, while confocal resolution and morphologic features are similar to histology [28]. This non-invasive imaging technique has been proven useful not only in the evaluation and follow-up of melanocytic [29,30] and non-melanocytic lesions [31,32,33,34,35,36,37], but also in the diagnosis of inflammatory skin diseases [38,39,40,41,42,43,44,45,46,47,48]. However, one fundamental risk of techniques such as RCM is that they rely on morphology-based analysis, thus being subject to interpretation bias. Previous studies addressing this subject exist [49,50], however, there is still a need for more research performed in accordance with the Standards of Reporting of Diagnostic Accuracy (STARD) [51].

The primary objective of this study was to determine the agreement between RCM and histopathology in correctly detecting BCC presence. The secondary objectives were to assess the accuracy of predefined confocal criteria in correctly classifying BCC histopathological subtypes and evaluate the intraobserver reliability of preoperative, non-invasive BCC diagnosis through RCM.

## 2. Materials and Methods

A retrospective multicenter study was performed at the following 2 sites: the Dermatology Research Laboratory, “Carol Davila” University of Medicine and Pharmacy, Bucharest, Romania and the Department of Dermatology at Medas Medical Center, Bucharest, Romania. Patient data was collected retrospectively by searching the electronic archives of the participating centers for patients registered between 1 May 2017 and 31 October 2018.

We included consecutively identified patients older than 18 years with a clinical and dermoscopic suspicion of previously untreated BCC, whose medical records included medical history, clinical, dermoscopic, and RCM images as well as a histopathologic report of the excisional biopsy of the lesion. We excluded patients with missing or incomplete data, patients with lesions that were reported to be recurrences, previously treated lesions, or lesions extending to mucosal surfaces. Immunocompromised patients were not excluded from the study. The study was conducted in accordance with the Declaration of Helsinki, and the protocol was approved by the Ethics Committee of the “Carol Davila” University of Medicine and Pharmacy Bucharest (Project Number 185, approved on 26.12.2018). All participants gave written informed consent as part of their investigation and treatment procedures, at the time of their registration.

In both centers, RCM examination was conducted using the same commercially available confocal microscope (VivaScope 1500^®^; Caliber ID, Henrietta, NY, USA.; MAVIG GmbH, München, Germany). RCM imaging at the Medas Medical Center was performed by ML and by CC at the “Prof. N. Paulescu” National Institute of Diabetes, Nutrition, and Metabolic Diseases.

The VivaScope 1500 uses an 830 nm laser diode, reaching a maximum output power of 20 mW at the skin level, allowing for skin imaging without causing injury to investigated tissues. A dermoscopic image captured using the VivaCam serves as a surface map to guide confocal imaging. Five level cubes (30 µm increments, vertically), including the corneal, granular/spinous, dermal-epidermal junction, and papillary dermis, are acquired in the center of the lesion. Each level is a mosaic with a minimum surface of 4 × 4 mm and a maximum of 8 × 8 mm. Individual stacks (4.5 µm increments, vertically) are also acquired in one or more areas of interest, up to a depth of 200 to 250 µm. Individual images of cellular and tissular architecture are also obtained. Only patients with lesions investigated following this RCM imaging protocol were included in the study. Verification that the RCM image set respects protocol was done through an inspection of the log file generated for each confocal examination.

Prior to the study, ML was trained in RCM use and interpretation during a one week confocal laser scanning microscopy course organized by MAVIG GmbH (distributor of the VivaScope^®^ device) at the University of Modena and Reggio Emilia in Italy. ML had more than three years of RCM experience prior to the start of the study. CC had more than nine years RCM experience.

Based on previous findings [49,50], a set of 14 confocal imaging criteria was formulated: keratinocyte atypia, epidermal streaming, ulceration, cords connected to the epidermis, small tumor islands (diameter <300 μm), large tumor islands (diameter >300 μm), hyporefractile silhouettes, peripheral palisading, clefting, increased vascularization, “onion-like” structures (corresponding to milia-like cysts), peritumoral collagen bundles, inflammation represented by bright dots and plump bright cells (corresponding to lymphocytes and melanophages), and dendritic cells inside tumor islands (corresponding to melanocytes). 

Imaging, at the time of patient evaluation, was not conducted in a blinded fashion as patient history and clinical examination had to be conducted as part of the standard clinical care. However, the database of static RCM images was analyzed in a blinded fashion by ML immediately after completion and locking, and four weeks after, to document the presence or absence of BCC and of the aforementioned criteria.

All lesions included in the study were surgically treated with margins between 3 to 5 mm. Histopathologic confirmation of BCC presence and subtype, and excision margins inspection using hematoxylin and eosin stained bread-loafed sections was defined as the reference standard. The reporting of histopathological findings was performed by experienced pathologists. During assessment of the reference standard, the pathologist was masked to the findings of the RCM examination, but not to the clinical description of the lesions and patients’ clinical history.

We recorded the following characteristics of participants and tumors and summarized them with descriptive statistics: age, gender, tumor topographic location, and tumor histopathological subtype. A distinction was made between superficial, nodular, and aggressive (micronodular, infiltrative, and basosquamous) BCC growth patterns. For the purposes of this study sclerodermiform/morpheaform BCC was considered equivalent to infiltrative BCC. In the case of mixed-type histopathological diagnosis, defined as two or more growth patterns, the most aggressive component was taken into account for analysis. 

The primary objective was the agreement between the index test (RCM) and reference standard (histopathology of the excision specimen) in correctly determining BCC presence. The secondary outcomes were estimating the accuracy of predefined confocal criteria in correctly classifying BCC subtypes and determining the intraobserver agreement of preoperative BCC diagnosis through RCM.

One rater (ML) reviewed all RCM images of de-identified cases twice, at a four-week interval. The rater was blinded to clinical and dermoscopic images, histopathological report, and to his previous interpretation. Between evaluations, RCM case numbers were shuffled and recoded by an online software-based algorithm (available at https://www.graphpad.com/quickcalcs/) to prevent identification. Evaluation data were recorded in a standardized manner to BCC presence (yes or no) and presence of the 14 selected criteria (yes or no).

Lesions in which subsequent surgical excision was not performed (reasons were recorded) were excluded. According to Shinkins et al. [52], the ideal approach to including and analyzing inconclusive valid test results (*n* = 8) is to treat them as if in a clinical scenario. We have, therefore, considered these inconclusive valid results as RCM positive cases, and included the cases which had received the reference standard (*n* = 2) in the analysis. The numbers of true and false positives and negatives were recorded. We established the sensitivity, specificity, positive, and negative likelihood ratios, and positive and negative predictive values for BCC diagnosis by RCM using 2 × 2 contingency tables analysis. To calculate the overall diagnostic accuracy, the following formula was used: Overall diagnostic accuracy = sensitivity × prevalence + specificity × (1 − prevalence) [53,54]. We used binomial logistic regression to determine the odds ratio (OR) of the predefined confocal criteria for each individual BCC histological subtype. Confidence intervals were 95% and a *p* value of <0.05 was considered significant. Intraobserver agreement was defined as the degree to which the assessment of selected RCM images is identic for repeated measurements by the same person on different occasions [55]. Cohen’s kappa was used to describe intraobserver agreement. Statistical analysis was performed using SPSS version 22.0 (IBM, New York, USA).

## 3. Results

### 3.1. Participants

**Figure 1:**
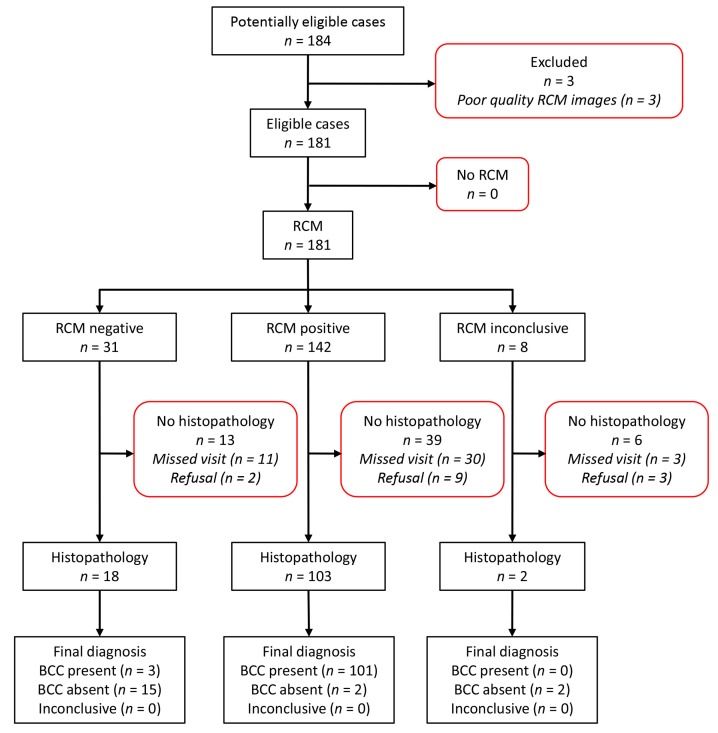
Diagram showing case selection process. RCM: reflectance confocal microscopy; BCC: basal cell carcinoma.

An electronic database search and chart review from the two participating centers identified 184 potentially eligible BCC cases. After evaluating each case for inclusion and exclusion criteria, we excluded three cases due to the poor quality of RCM images. Out of the 181 eligible BCC cases, 58 had not received the index test or the reference standard, hence they were excluded. The two inconclusive RCM cases with histopathological analysis were treated as test positives, leaving a total number of 123 lesions from 87 patients for further analysis (Figure 1).

Eighty-seven patients (36 males and 51 females) with a mean age of 68.1 ± 12.17 years and median disease duration of 2 years were included in the study. Most lesions were of the nodular subtype, with 11 aggressive BCCs (aBCCs) represented (7 infiltrative BCCs, 3 basosquamous BCCs, and one micronodular BCC), which is consistent with the natural incidence of BCC subtypes. The distribution of the 104 BCCs in terms of subtype and the histopathological diagnoses for the remaining 19 lesions are summarized in Table 1.

Most lesions were located in the head and neck area (*n* = 72), followed by the trunk (*n* = 30), lower extremities (*n* = 10), upper extremities (*n* = 8), and abdomen (*n* = 3). The number of BCCs in our study (*n* = 104) is sufficient to confidently calculate sensitivity and specificity with a maximum error of estimation of 6% and 14.1%, respectively, with a confidence interval of 1-alpha = 0.95 (95%). The average time between RCM examination and surgical treatment was 50.99 days.

### 3.2. Test Results

#### 3.2.1. Basal Cell Carcinoma Diagnosis by Preoperative Reflectance Confocal Microscopy

In our sample of 123 lesions, RCM detected BCC presence with a sensitivity of 97.1% (95% CI 91.80, 99.40) and a specificity of 78.95% (95% CI 54.43, 93.95) at a disease prevalence of 84.55%. The positive likelihood ratio was 4.61 (95% CI 1.93, 11.03) while the negative likelihood ratio was 0.04 (95% CI 0.01, 0.11). Positive and negative predictive values were 96.19% (95% CI 91.35, 98.37) and 83.33% (95% CI 61.55, 93.98), respectively. The overall accuracy of preoperative RCM for detection of BCC was 94.31% (95% CI 88.63, 97.68).

If only conclusive RCM analysis results were included in the analysis (*n* = 121), RCM sensitivity was unchanged at 97.1% (95% CI 91.80, 99.40), but specificity was higher at 88.2% (95% CI 63.56, 98.54), as was disease prevalence (85.95%). The positive likelihood ratio was 8.25 (95% CI 2.24, 30) while the negative likelihood ratio was 0.03 (95% CI 0.01, 0.1). Positive and negative predictive values were 98.1% (95% CI 93.21, 99.46) and 83.3% (95% CI 61.79, 93.92), respectively. The overall accuracy of preoperative RCM for detection of BCC in this case was only slightly higher, at 95.87% (95% CI 90.62, 98.64). 

#### 3.2.2. Evaluation of RCM Criteria According to BCC Subtype

**Figure 2:**
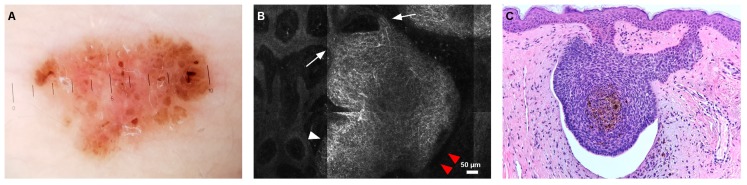
Superficial basal cell carcinoma. (**A**) Dermoscopy showing multiple, brown globules and dots, and leaf-like peripheral structures. (**B**) reflectance confocal microscopy (RCM) revealed the presence of sharply demarcated cords connected to the epidermis (white arrows), dark peritumoral clefting (red arrowheads), and peripheral palisading (white arrowhead), corresponding to the basaloid cords and aggregates seen on histopathology (hematoxylin and eosin (H&E) stain, magnification 4×) (**C**).

In superficial BCCs (sBCCs), RCM examination revealed the presence of cords connected to the epidermis (13/24) with peripheral palisading (19/24) (Figure 2). 

**Figure 3:**
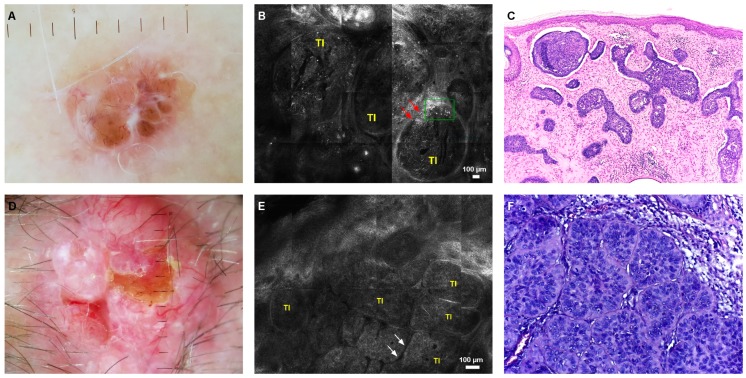
Nodular basal cell carcinoma. (**A**) Dermoscopy image of a pigmented nodular basal cell carcinoma (nBCC) with blue-gray ovoid nests, brown globules, and arborizing vessels. (**B**) RCM revealed large, well defined tumor islands (TI), peritumoral clefting (red arrows), and clumped melanophages (green rectangle). (**C**) Histopathology showed large basaloid islands with palisading and stromal retraction in the dermis (H&E stain, magnification 4×). (**D**) Dermoscopy of hypopigmented nBCC with ulceration and arborizing vessels. (**E**) RCM showed large tumor islands (TI) with peripheral palisading and clefting (white arrows). (**F**) Histopathologic correlates of the structures seen through RCM (H&E stain, magnification 40×).

Moreover, dendritic structures inside tumor islands and cords (14/24) were frequently seen. For nodular BCCs (nBCCs), big tumor islands (52/69) associated with peripheral palisading (42/69), clefting (34/69), and hypervascularization (52/69) were characteristic findings (Figure 3).

**Figure 4:**
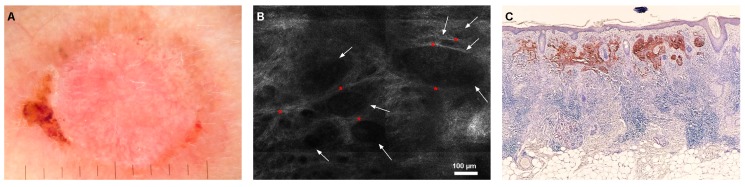
Infiltrative basal cell carcinoma. (**A**) Dermoscopy showing a hypopigmented lesion with structureless red shiny areas, chrysalis pattern, short fine telangiectasia, and erosion. (**B**) RCM showed multiple hyporefractile silhouettes, which appear as imprints (white arrows) outlined by bundles of bright collagen (red asterisks). (**C**) Histopathology shows tumor islands and strands that resemble the hyporefractile silhouettes observed through RCM (BerEP4 stain, magnification 4×).

Aggressive BCCs were typified by the presence of hyporefractile silhouettes (7/11), peripheral palisading (7/11), and clefting (7/11) (Figure 4). 

Keratinocyte atypia, epidermal streaming, ulceration, and inflammation were observed with comparable frequencies in all tumor subtypes. The analytic descriptive results of the confocal image analysis are summarized in Table 2.

#### 3.2.3. Logistic Regression Analysis for RCM Criteria in BCC Subtyping

We used both univariate and multivariate logistic regression to model the influence of RCM criteria on BCC subtype classification (odds ratios in Table 3 correspond to each BCC subtype).

In univariate analysis, nBCC was almost six times more likely (OR = 5.863, 95% CI (2.415, 14.236), *p* < 0.01) if big tumor islands had been observed, and more than six times more likely if peritumoral collagen bundles were present (OR = 6.703, 95% CI (2.136, 21.036), *p* = 0.01). Superficial BCC was almost 14 times more likely if cords connected to the epidermis had been observed (OR = 13.636, 95% CI (4.247, 43.784), *p* < 0.001). The presence of hyporefractile silhouettes was associated with five-fold higher odds for aBCC (OR = 5.648, 95% CI (1.511, 21.105), *p* = 0.01). 

We entered all 14 RCM criteria in a multivariate logistic regression analysis with backward elimination according to likelihood ratios and a classification cutoff of 0.5. Three separate models were created, one for each BCC subtype. The nBCC model correctly classified 66.3% of cases before including regression criteria and 81.7% after adding predictors, gaining a substantial increase in percentage accuracy in classification (PAC). Nodular BCC was more likely in the presence of peritumoral collagen bundles (OR = 11.454, 95% CI (1.636, 80.188), *p* = 0.014), increased vascularization (OR = 4.359, 95% CI (1.071, 17.730), *p* = 0.04), and if cords connected to the epidermis were absent (10.41 times lower odds; *p* = 0.008). For sBCC, the constant model correctly classified 77.9% of cases and 87.5% with the predictors added. Superficial BCC was the most common diagnosis if cords connected to the epidermis were observed (6.794-fold higher odds; *p* = 0.017). For aBCC, the change in PAC after adding the RCM criteria was smaller (1%). Aggressive BCC was most common in the presence of hyporefractile silhouettes (OR = 16.92, 95% CI (1.915, 149.499), *p* = 0.01) and the absence of big tumor islands (4.4-fold lower odds; *p* = 0.048). 

Even though big tumor islands and peritumoral collagen bundles were strongly associated with nBCC in the univariate analysis, this effect was diminished by the influence of other variables in the multivariate statistical model. In aBCC, hyporefractile silhouettes remained a potent predictor in the univariate, but even more so in the multivariate model.

#### 3.2.4. Intraobserver Agreement

The intraobserver agreement for BCC presence calculated from the cross-tabulation was 97.56%. Cohen’s kappa was run to determine the intraobserver agreement between the two evaluations. The analysis showed that there was excellent agreement between the two evaluations, κ = 0.909 (95% CI 0.807, 1), *p* < 0.001.

#### 3.2.5. Adverse Events for Index Test and Reference Standard

There were no adverse events after performing RCM. Adverse reactions after surgical excision included five patients with post-operative wound infections. All cases were successfully treated with oral antibiotics, without the need of hospitalization. There were no serious adverse reactions.

## 4. Discussion

Previous studies assessing RCM for BCC diagnosis report varying sensitivity and specificity values ranging from 85%–97% and 89%–99%, respectively [56]. Our results confirm the high sensitivity (97.1%) and specificity (78.95%) of RCM for diagnosing BCC.

There is considerable dermoscopic pattern variability among different BCC histologic subtypes and, recently, dermoscopy has been shown to accurately discriminate between superficial BCC and all other histopathologic subtypes in approximately 80% of cases, based on a series of criteria [24], meaning that the remaining 20% of tumors that do not fit these criteria require histopathologic examination for subtyping. However, this study included only histopathologically proven BCCs, thus the validity of the criteria for differentiating BCC from other diseases was not assessed. Furthermore, differences in the incidence and frequency of various BCC subtypes among different populations should be taken into account [57].

Reflectance confocal microscopic criteria associated with BCC have been previously described by several authors, with tumor islands and cords being considered as this tumor’s trademark [50,58,59,60,61,62,63,64,65,66].

Our study reveals significant differences in the confocal patterns among BCC subtypes, confirming that RCM provides additional morphologic information and suggesting that RCM enhances the preoperative diagnosis of BCC as well as its subtype classification. This aspect is particularly important in clinical practice since the therapeutic approach of BCC is largely determined by its histopathological subtype.

Our findings confirm previous reported data on RCM findings in BCC and associates specific criteria with different BCC subtypes. First and foremost, tumor cords connected to the epidermis strongly and significantly predicted sBCC, thus supporting previous data [49]. Epidermal streaming is described as one of the most important RCM criterion for the diagnosis of BCC [50,67], however, in our study, epidermal streaming was found in only 37.7% of sBCCs and was not statistically significant in predicting histotype. This result is in accordance with a previous study [49], which reports a 50% frequency of epidermal streaming in sBCC and 50% increased odds of sBCC, although without statistical significance. This may be connected to, as the authors have observed, the increased degree of subjectivity that comes into play when assessing this parameter. Nodular BCC was typified by the presence of big tumor islands and peritumoral collagen bundles, confirming the findings of Longo et al. [49]. Although increased vascularization was detected in all tumoral subtypes in our dataset, in multivariate analysis, this parameter was a predictor only for nBCC. In Nori et al.’s study [50], increased vascularization had a sensitivity of 83.9% and 95.7% and a specificity of 53.6% and 53.6% for nodular and superficial BCC, respectively. A cord connected to the epidermis was, in our study, a negative predictor for nBCC, their absence resulting in 10-times lower odds for this subtype. Previous results also show 93% lower odds for nBCC in the presence of cords connected to the epidermis [49]. Aggressive BCC was characterized by the presence of hyporefractile silhouettes (63.6%), while others have found these structures in 77.3% of infiltrative BCCs [49]. Furthermore, aBCC was the most common diagnosis in the absence of big tumor islands, a finding corroborated by others [49]. However, due to the particular appearance of hyporefractile silhouettes, their recognition might require substantial experience with confocal microscopy. Histopathologically, these structures correspond to non-pigmented tumor islands. While previous studies [49] report a high frequency (95.5%) of collagen fibers surrounding tumor islands in infiltrative BCCs, although without statistical significance, in our study, this criterion was present in only 36.4% of aBCCs and was not a statistically significant predictor.

Keratinocyte atypia and inflammation were present in the majority of tumors of all subtypes, while ulceration, onion-like structures, and dendritic structures inside tumor islands were less frequently seen, results also confirmed by previous findings [49,66]. Nori et al. [50] found the sensitivity of pleomorphic epidermis (keratinocyte atypia) for sBCC and nBCC to be 56.5% and 65.5%, while specificity was 63.8% for both subtypes. 

In our study, the intraobserver agreement was assessed based on static de-identified RCM images. We report an intraobserver agreement for BCC presence of 97.56%, thus confirming previous findings of the reliability of RCM in correctly and consistently diagnosing BCC [56,68]. However, we believe this simple method of assessing agreement is flawed because it does not take into account chance agreement [69]. Therefore, Cohen’s kappa was run, showing excellent agreement (κ = 0.909, 95% CI (0.807, 1), *p* < 0.001). We consider this to be one of the strengths of our study, along with the adherence to the STARD guidelines [51]. Using predefined RCM criteria and the same generation VivaScope 1500 device at both participating centers helped prevent heterogeneity of the results.

Limitations of our study include the retrospective design, which is subject to recall and observer bias. These have been addressed by the use of de-identified RCM images and the shuffling of images between evaluations. The use of static RCM images brings external validity issues into discussion, as there are significant differences between diagnosing and subtyping BCCs using blinded static images and real-time RCM combined with clinical information and dermoscopy [70]. We believe that in a real clinical scenario, where real-time RCM is typically used as an adjunct imaging tool to patient history, clinical examination, and dermoscopy, diagnostic accuracy measures of this complementary approach could be even higher. However, this assumption needs to be corroborated through further, preferably prospective, studies. Secondly, our study included a limited number of patients and our findings need to be confirmed prospectively to more precisely determine the sensitivity and specificity of these diagnostic criteria. Thirdly, our sample did not include any melanomas, one of the biggest differential diagnostic concerns of clinicians evaluating skin tumors, making this another limitation. Two of the biggest challenges RCM users face in accurately discriminating between BCC subtypes are the relative lack of studies reporting the reliability of subtype-specific confocal criteria and the limited depth of imaging of the RCM device (approximately 200–250 μm). The latter is already being addressed by several ongoing studies [71,72].

The use of RCM to avoid skin biopsy in selected cases could lead to a significant cost reduction if we consider that RCM requires one user and one confocal imaging device, while a skin biopsy necessitates a minimum of four persons: dermatologist, nurse, histopathology laboratory technician, and pathologist. However, prospective cost-effectiveness studies of RCM versus skin biopsy should be conducted in order to determine if there is a financial advantage to be gained. Previous diagnostic RCM studies have focused on sensitivity and specificity for diagnosing BCC and its histopathological subtypes, however, other aspects, such as time between diagnosis and treatment, should also be considered. The average time period between RCM and surgery in our study was 50.99 days, although this was due mostly to patient related factors. In our experience, RCM imaging only takes about 10 to 15 min per lesion, therefore optimizing patient flow from presentation to the operating room. Thus, one of the main advantages using RCM is on the spot diagnosis and treatment of BCCs compared to painful procedures, such as skin biopsies, with all the delays this implies. In the future, RCM could potentially replace the skin biopsy before Mohs micrographic surgery procedures, saving time, funds, and an avoidable and painful procedure. Moreover, by using the more flexible hand-held VivaScope 3000 (VivaScope 3000; Caliber ID, Henrietta, NY, USA.), clinically suspicious lesions can be evaluated even faster. Moreover, selected cases of sBCC patients could potentially benefit from completely non-invasive management [73]. 

## 5. Conclusions

In conclusion, our study shows that RCM is reliable in correctly diagnosing BCC and identifies specific confocal criteria associated with BCC subtypes. If accurate subtyping is achieved, RCM could play a key role in BCC management, therefore additional prospective studies are required to investigate whether the combination of dermoscopy and RCM would help increase the accuracy of preoperative BCC subtype classification. 

## Figures and Tables

**Table 1:** Characteristics of patients and imaged lesions.

**BCC ^1^ Subtype**	***N* (%)**
Superficial BCC	24 (23.1)
Nodular BCC	69 (66.3)
Aggressive BCC	11 (10.6)
Total = 104	
**Non-BCC Lesions**	***N* (%)**
Bowen’s disease	3 (2.4)
Seborrheic keratosis	3 (2.4)
Actinic keratosis	4 (3.3)
Keratoacanthoma	2 (1.6)
Lichen planus-like keratosis	2 (1.6)
Tubular apocrine adenoma	1 (0.8)
Moderately differentiated SCC ^2^	1 (0.8)
Poorly differentiated SCC	1 (0.8)
Poroid hidradenoma	1 (0.8)
Chronic radiation dermatitis	1 (0.8)
Total = 19	

BCC ^1^, basal cell carcinoma; SCC ^2^, squamous cell carcinoma.

**Table 2:** Frequencies of confocal criteria in different histologic basal cell carcinoma subtypes.

Confocal Criterion, *N* (%)	BCC ^1^ Histologic Subtype		
	Nodular (*N* = 69)	Superficial (*N* = 24)	Aggressive (*N* = 11)
Keratinocyte atypia	49 (71)	17 (70.8)	10 (90.9)
Epidermal streaming	21 (30.4)	9 (37.5)	5 (45.5)
Ulceration	24 (34.8)	5 (20.8)	4 (36.4)
Cords connected to the epidermis	3 (4.3)	13 (54.2)	2 (18.2)
Small tumor islands	25 (36.2)	3 (12.5)	6 (54.5)
Big tumor islands	52 (75.4)	8 (33.3)	4 (36.4)
Hyporefractile silhouettes	21 (30.4)	1 (4.2)	7 (63.6)
Peripheral palisading	42 (60.9)	19 (79.2)	7 (63.6)
Clefting	34 (49.3)	11 (45.8)	7 (63.6)
Increased vascularization	52 (75.4)	8 (33.3)	4 (36.4)
Onion-like structures	22 (31.9)	3 (12.5)	5 (45.5)
Peritumoral collagen bundles	32 (46.4)	0 (0)	4 (36.4)
Inflammation	58 (84.1)	19 (79.2)	9 (81.8)
Dendritic structures inside tumor islands	35 (50.7)	14 (58.3)	4 (36.4)

BCC ^1^, basal cell carcinoma.

**Table 3:** Multivariate reflectance confocal microscopy criteria predictors for nodular, superficial, and aggressive subtypes of basal cell carcinoma.

	*p* Value	OR ^1^	95% CI ^2^ for OR
**Nodular**			
Collagen surrounding tumor islands	0.014	11.454	1.636–80.188
Increased vascularization	0.04	4.359	1.071–17.730
Cords connected to the epidermis	0.008	0.096	0.017–0.543
**Superficial**			
Cords connected to the epidermis	0.017	6.794	1.399–32.991
**Aggressive**			
Hyporefractile silhouettes	0.01	16.92	1.915–149.499
Big tumor islands	0.048	0.227	0.052–0.988

OR ^1^, Odds Ratio; CI ^2^, Confidence Interval.
